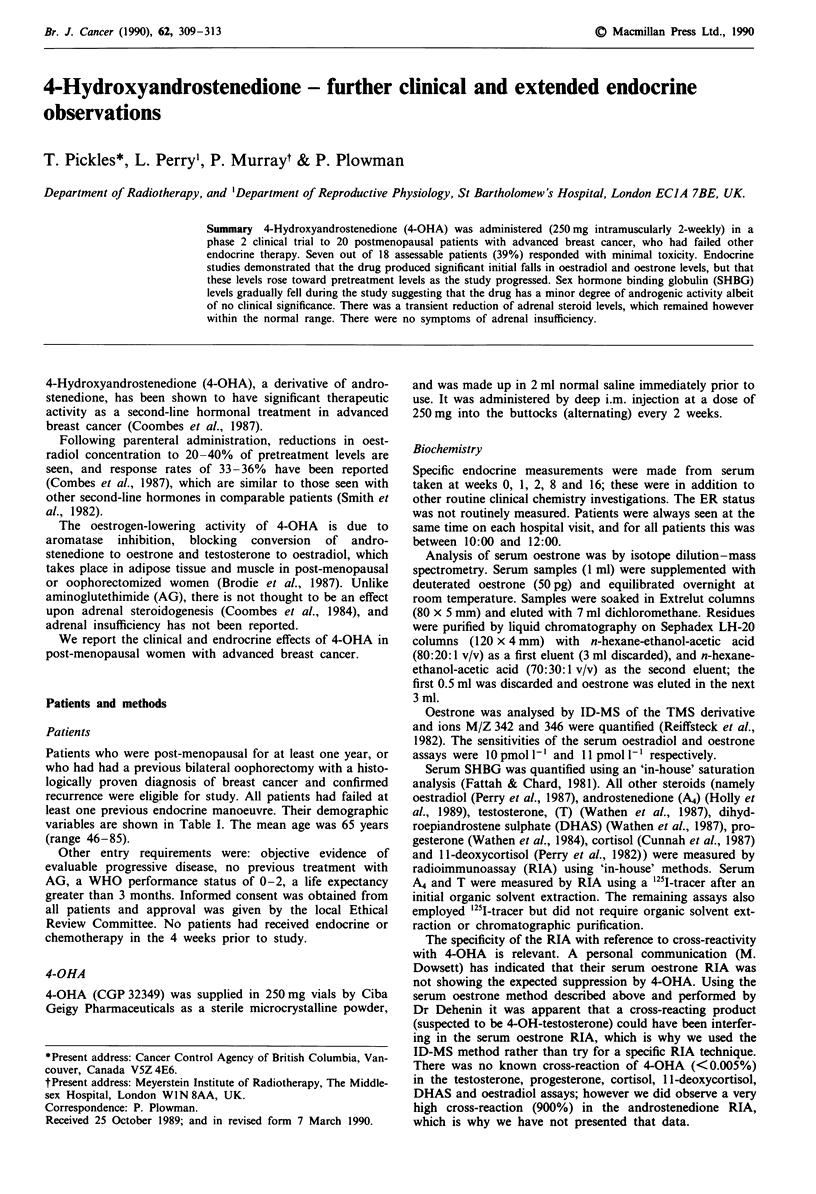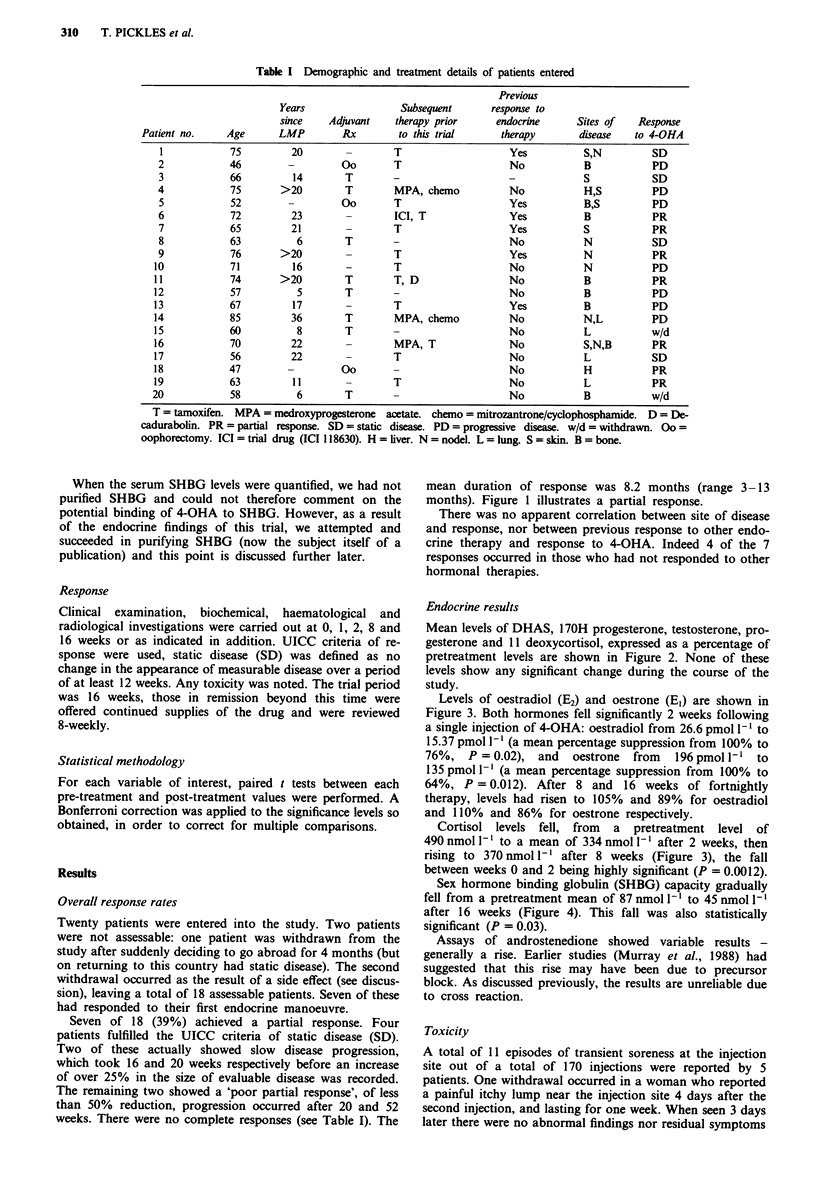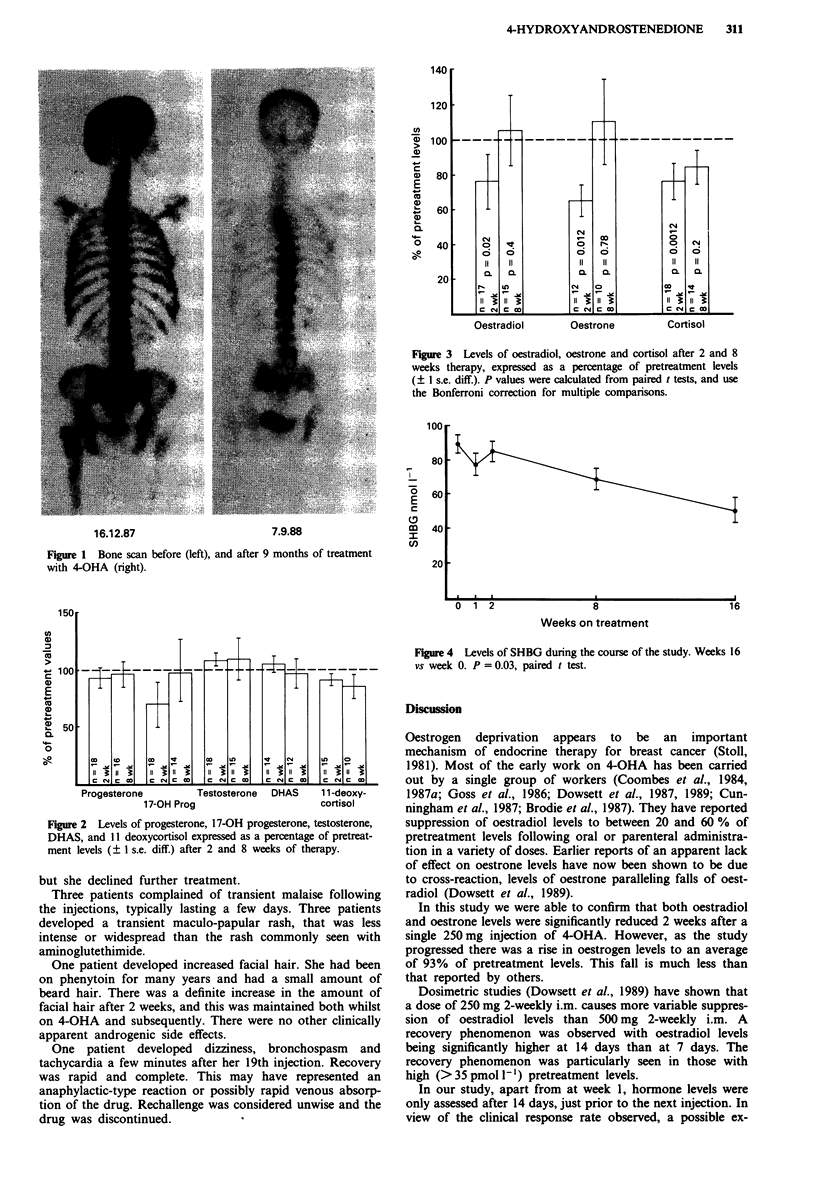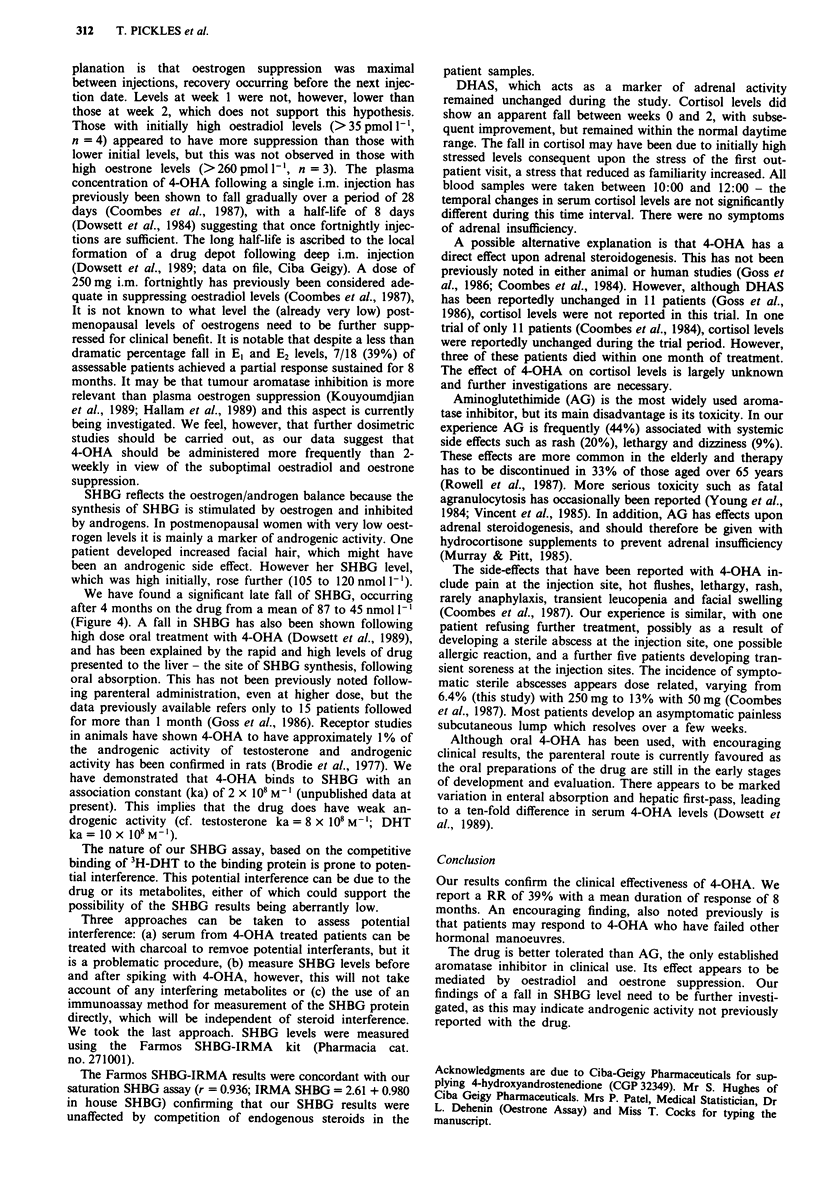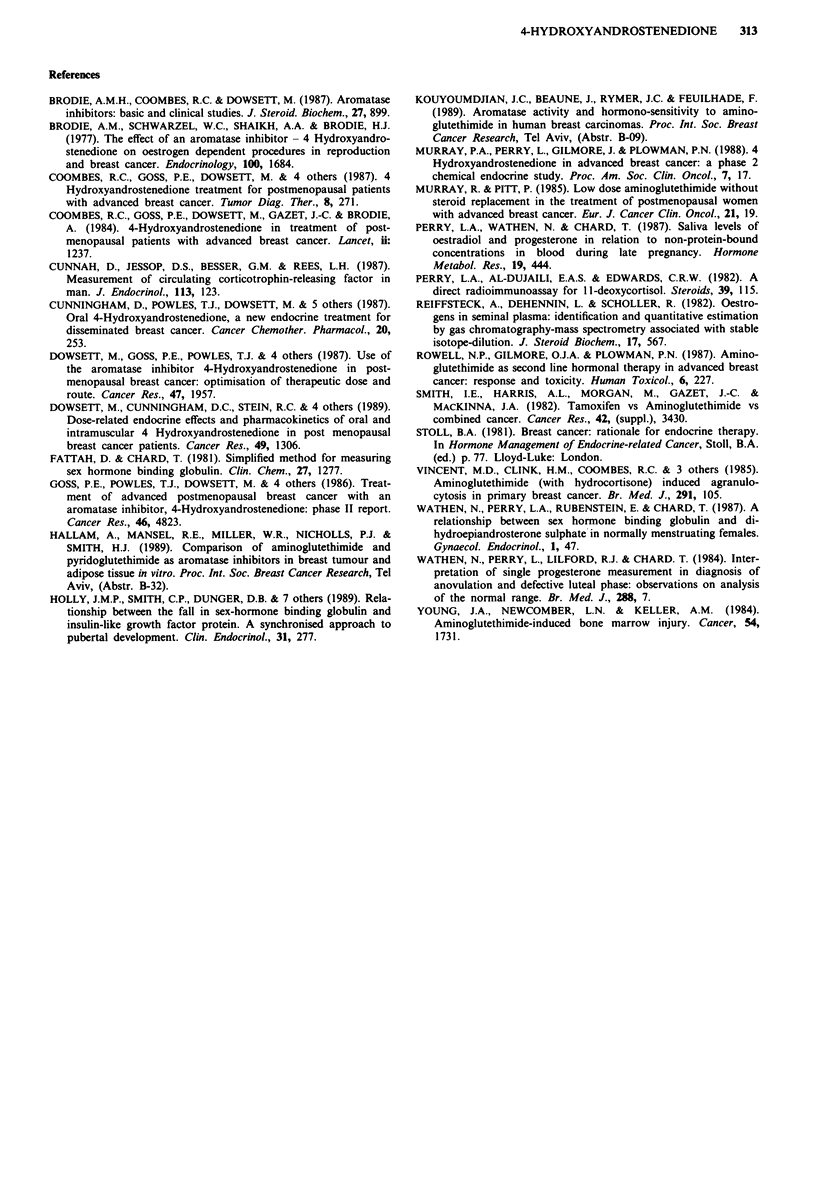# 4-hydroxyandrostenedione--further clinical and extended endocrine observations.

**DOI:** 10.1038/bjc.1990.284

**Published:** 1990-08

**Authors:** T. Pickles, L. Perry, P. Murray, P. Plowman

**Affiliations:** Department of Radiotherapy, St Bartholomew's Hospital, London, UK.

## Abstract

**Images:**


 © Macmillan Press Ltd., 1990

### 4-Hydroxyandrostenedione – further clinical and extended endocrine observations

T. Pickles*, L. Perry[1], P. Murray† & P. Plowman

*Department of Radiotherapy, and [1]Department of Reproductive Physiology, St Bartholomew's Hospital, London EC1A 7BE, UK.*

**Summary** 4-Hydroxyandrostenedione (4-OHA) was administered (250 mg intramuscularly 2-weekly) in a phase 2 clinical trial to 20 postmenopausal patients with advanced breast cancer, who had failed other endocrine therapy. Seven out of 18 assessable patients (39%) responded with minimal toxicity. Endocrine studies demonstrated that the drug produced significant initial falls in oestradiol and oestrone levels, but that these levels rose toward pretreatment levels as the study progressed. Sex hormone binding globulin (SHBG) levels gradually fell during the study suggesting that the drug has a minor degree of androgenic activity albeit of no clinical significance. There was a transient reduction of adrenal steroid levels, which remained however within the normal range. There were no symptoms of adrenal insufficiency.

4-Hydroxyandrostenedione (4-OHA), a derivative of androstenedione, has been shown to have significant therapeutic activity as a second-line hormonal treatment in advanced breast cancer (Coombes *et al.*, 1987).

Following parenteral administration, reductions in oestradiol concentration to 20–40% of pretreatment levels are seen, and response rates of 33–36% have been reported (Combes *et al.*, 1987), which are similar to those seen with other second-line hormones in comparable patients (Smith *et al.*, 1982).

The oestrogen-lowering activity of 4-OHA is due to aromatase inhibition, blocking conversion of androstenedione to oestrone and testosterone to oestradiol, which takes place in adipose tissue and muscle in post-menopausal or oophorectomized women (Brodie *et al.*, 1987). Unlike aminoglutethimide (AG), there is not thought to be an effect upon adrenal steroidogenesis (Coombes *et al.*, 1984), and adrenal insufficiency has not been reported.

We report the clinical and endrocrine effects of 4-OHA in post-menopausal women with advanced breast cancer.

### Patients and methods

### *Patients*

Patients who were post-menopausal for at least one year, or who had had a previous bilateral oophorectomy with a histologically proven diagnosis of breast cancer and confirmed recurrence were eligible for study. All patients had failed at least one previous endocrine manoeuvre. Their demographic variables are shown in Table I. The mean age was 65 years (range 46–85).

Other entry requirements were: objective evidence of evaluable progressive disease, no previous treatment with AG, a WHO performance status of 0–2, a life expectancy greater than 3 months. Informed consent was obtained from all patients and approval was given by the local Ethical Review Committee. No patients had received endocrine or chemotherapy in the 4 weeks prior to study.

### *4-OHA*

4-OHA (CGP 32349) was supplied in 250 mg vials by Ciba Geigy Pharmaceuticals as a sterile microcrystalline powder, and was made up in 2 ml normal saline immediately prior to use. It was administered by deep i.m. injection at a dose of 250 mg into the buttocks (alternating) every 2 weeks.

### *Biochemistry*

Specific endocrine measurements were made from serum taken at weeks 0, 1, 2, 8 and 16; these were in addition to other routine clinical chemistry investigations. The ER status was not routinely measured. Patients were always seen at the same time on each hospital visit, and for all patients this was between 10:00 and 12:00.

Analysis of serum oestrone was by isotope dilution–mass spectrometry. Serum samples (1 ml) were supplemented with deuterated oestrone (50 pg) and equilibrated overnight at room temperature. Samples were soaked in Extrelut columns (80 × 5 mm) and eluted with 7 ml dichloromethane. Residues were purified by liquid chromatography on Sephadex LH-20 columns (120 × 4 mm) with *n*-hexane-ethanol-acetic acid (80:20:1 v/v) as a first eluent (3 ml discarded), and *n*-hexane-ethanol-acetic acid (70:30:1 v/v) as the second eluent; the first 0.5 ml was discarded and oestrone was eluted in the next 3 ml.

Oestrone was analysed by ID-MS of the TMS derivative and ions M/Z 342 and 346 were quantified (Reiffsteck *et al.*, 1982). The sensitivities of the serum oestradiol and oestrone assays were 10 pmol $l^{-1}$ and 11 pmol $l^{-1}$ respectively.

Serum SHBG was quantified using an 'in-house' saturation analysis (Fattah & Chard, 1981). All other steroids (namely oestradiol (Perry *et al.*, 1987), androstenedione ($A_4$) (Holly *et al.*, 1989), testosterone, (T) (Wathen *et al.*, 1987), dihydroepiandrostene sulphate (DHAS) (Wathen *et al.*, 1987), progesterone (Wathen *et al.*, 1984), cortisol (Cunnah *et al.*, 1987) and 11-deoxycortisol (Perry *et al.*, 1982)) were measured by radioimmunoassay (RIA) using 'in-house' methods. Serum $A_4$ and T were measured by RIA using a $^{125}$I-tracer after an initial organic solvent extraction. The remaining assays also employed $^{125}$I-tracer but did not require organic solvent extraction or chromatographic purification.

The specificity of the RIA with reference to cross-reactivity with 4-OHA is relevant. A personal communication (M. Dowsett) has indicated that their serum oestrone RIA was not showing the expected suppression by 4-OHA. Using the serum oestrone method described above and performed by Dr Dehenin it was apparent that a cross-reacting product (suspected to be 4-OH-testosterone) could have been interfering in the serum oestrone RIA, which is why we used the ID-MS method rather than try for a specific RIA technique. There was no known cross-reaction of 4-OHA (<0.005%) in the testosterone, progesterone, cortisol, 11-deoxycortisol, DHAS and oestradiol assays; however we did observe a very high cross-reaction (900%) in the androstenedione RIA, which is why we have not presented that data.

*Present address: Cancer Control Agency of British Columbia, Vancouver, Canada V5Z 4E6.
†Present address: Meyerstein Institute of Radiotherapy, The Middlesex Hospital, London W1N 8AA, UK.
Correspondence: P. Plowman.
Received 25 October 1989; and in revised form 7 March 1990.

**Table I** Demographic and treatment details of patients entered

| Patient no. | Age | Years since LMP | Adjuvant Rx | Subsequent therapy prior to this trial | Previous response to endocrine therapy | Sites of disease | Response to 4-OHA |
|---|---|---|---|---|---|---|---|
| 1 | 75 | 20 | – | T | Yes | S,N | SD |
| 2 | 46 | – | Oo | T | No | B | PD |
| 3 | 66 | 14 | T | – | – | S | SD |
| 4 | 75 | >20 | T | MPA, chemo | No | H,S | PD |
| 5 | 52 | – | Oo | T | Yes | B,S | PD |
| 6 | 72 | 23 | – | ICI, T | Yes | B | PR |
| 7 | 65 | 21 | – | T | Yes | S | PR |
| 8 | 63 | 6 | T | – | No | N | SD |
| 9 | 76 | >20 | – | T | Yes | N | PR |
| 10 | 71 | 16 | – | T | No | N | PD |
| 11 | 74 | >20 | T | T, D | No | B | PR |
| 12 | 57 | 5 | T | – | No | B | PD |
| 13 | 67 | 17 | – | T | Yes | B | PD |
| 14 | 85 | 36 | T | MPA, chemo | No | N,L | PD |
| 15 | 60 | 8 | T | – | No | L | w/d |
| 16 | 70 | 22 | – | MPA, T | No | S,N,B | PR |
| 17 | 56 | 22 | – | T | No | L | SD |
| 18 | 47 | – | Oo | – | No | H | PR |
| 19 | 63 | 11 | – | T | No | L | PR |
| 20 | 58 | 6 | T | – | No | B | w/d |

T = tamoxifen. MPA = medroxyprogesterone acetate. chemo = mitrozantrone/cyclophosphamide. D = Decadurabolin. PR = partial response. SD = static disease. PD = progressive disease. w/d = withdrawn. Oo = oophorectomy. ICI = trial drug (ICI 118630). H = liver. N = nodel. L = lung. S = skin. B = bone.

When the serum SHBG levels were quantified, we had not purified SHBG and could not therefore comment on the potential binding of 4-OHA to SHBG. However, as a result of the endocrine findings of this trial, we attempted and succeeded in purifying SHBG (now the subject itself of a publication) and this point is discussed further later.

### *Response*

Clinical examination, biochemical, haematological and radiological investigations were carried out at 0, 1, 2, 8 and 16 weeks or as indicated in addition. UICC criteria of response were used, static disease (SD) was defined as no change in the appearance of measurable disease over a period of at least 12 weeks. Any toxicity was noted. The trial period was 16 weeks, those in remission beyond this time were offered continued supplies of the drug and were reviewed 8-weekly.

### *Statistical methodology*

For each variable of interest, paired $t$ tests between each pre-treatment and post-treatment values were performed. A Bonferroni correction was applied to the significance levels so obtained, in order to correct for multiple comparisons.

### Results

### *Overall response rates*

Twenty patients were entered into the study. Two patients were not assessable: one patient was withdrawn from the study after suddenly deciding to go abroad for 4 months (but on returning to this country had static disease). The second withdrawal occurred as the result of a side effect (see discussion), leaving a total of 18 assessable patients. Seven of these had responded to their first endocrine manoeuvre.

Seven of 18 (39%) achieved a partial response. Four patients fulfilled the UICC criteria of static disease (SD). Two of these actually showed slow disease progression, which took 16 and 20 weeks respectively before an increase of over 25% in the size of evaluable disease was recorded. The remaining two showed a 'poor partial response', of less than 50% reduction, progression occurred after 20 and 52 weeks. There were no complete responses (see Table I). The mean duration of response was 8.2 months (range 3–13 months). Figure 1 illustrates a partial response.

There was no apparent correlation between site of disease and response, nor between previous response to other endocrine therapy and response to 4-OHA. Indeed 4 of the 7 responses occurred in those who had not responded to other hormonal therapies.

### *Endocrine results*

Mean levels of DHAS, 17OH progesterone, testosterone, progesterone and 11 deoxycortisol, expressed as a percentage of pretreatment levels are shown in Figure 2. None of these levels show any significant change during the course of the study.

Levels of oestradiol ($E_2$) and oestrone ($E_1$) are shown in Figure 3. Both hormones fell significantly 2 weeks following a single injection of 4-OHA: oestradiol from 26.6 pmol $l^{-1}$ to 15.37 pmol $l^{-1}$ (a mean percentage suppression from 100% to 76%, $P = 0.02$), and oestrone from 196 pmol $l^{-1}$ to 135 pmol $l^{-1}$ (a mean percentage suppression from 100% to 64%, $P = 0.012$). After 8 and 16 weeks of fortnightly therapy, levels had risen to 105% and 89% for oestradiol and 110% and 86% for oestrone respectively.

Cortisol levels fell, from a pretreatment level of 490 nmol $l^{-1}$ to a mean of 334 nmol $l^{-1}$ after 2 weeks, then rising to 370 nmol $l^{-1}$ after 8 weeks (Figure 3), the fall between weeks 0 and 2 being highly significant ($P = 0.0012$).

Sex hormone binding globulin (SHBG) capacity gradually fell from a pretreatment mean of 87 nmol $l^{-1}$ to 45 nmol $l^{-1}$ after 16 weeks (Figure 4). This fall was also statistically significant ($P = 0.03$).

Assays of androstenedione showed variable results – generally a rise. Earlier studies (Murray *et al.*, 1988) had suggested that this rise may have been due to precursor block. As discussed previously, the results are unreliable due to cross reaction.

### *Toxicity*

A total of 11 episodes of transient soreness at the injection site out of a total of 170 injections were reported by 5 patients. One withdrawal occurred in a woman who reported a painful itchy lump near the injection site 4 days after the second injection, and lasting for one week. When seen 3 days later there were no abnormal findings nor residual symptoms

**Figure 1** Bone scan before (left), and after 9 months of treatment with 4-OHA (right).

**Figure 2** Levels of progesterone, 17-OH progesterone, testosterone, DHAS, and 11 deoxycortisol expressed as a percentage of pretreatment levels (± 1 s.e. diff.) after 2 and 8 weeks of therapy.

but she declined further treatment.

Three patients complained of transient malaise following the injections, typically lasting a few days. Three patients developed a transient maculo-papular rash, that was less intense or widespread than the rash commonly seen with aminoglutethimide.

One patient developed increased facial hair. She had been on phenytoin for many years and had a small amount of beard hair. There was a definite increase in the amount of facial hair after 2 weeks, and this was maintained both whilst on 4-OHA and subsequently. There were no other clinically apparent androgenic side effects.

One patient developed dizziness, bronchospasm and tachycardia a few minutes after her 19th injection. Recovery was rapid and complete. This may have represented an anaphylactic-type reaction or possibly rapid venous absorption of the drug. Rechallenge was considered unwise and the drug was discontinued.

**Figure 3** Levels of oestradiol, oestrone and cortisol after 2 and 8 weeks therapy, expressed as a percentage of pretreatment levels (± 1 s.e. diff.). *P* values were calculated from paired *t* tests, and use the Bonferroni correction for multiple comparisons.

**Figure 4** Levels of SHBG during the course of the study. Weeks 16 *vs* week 0. $P = 0.03$, paired *t* test.

### Discussion

Oestrogen deprivation appears to be an important mechanism of endocrine therapy for breast cancer (Stoll, 1981). Most of the early work on 4-OHA has been carried out by a single group of workers (Coombes *et al.*, 1984, 1987*a*; Goss *et al.*, 1986; Dowsett *et al.*, 1987, 1989; Cunningham *et al.*, 1987; Brodie *et al.*, 1987). They have reported suppression of oestradiol levels to between 20 and 60 % of pretreatment levels following oral or parenteral administration in a variety of doses. Earlier reports of an apparent lack of effect on oestrone levels have now been shown to be due to cross-reaction, levels of oestrone paralleling falls of oestradiol (Dowsett *et al.*, 1989).

In this study we were able to confirm that both oestradiol and oestrone levels were significantly reduced 2 weeks after a single 250 mg injection of 4-OHA. However, as the study progressed there was a rise in oestrogen levels to an average of 93% of pretreatment levels. This fall is much less than that reported by others.

Dosimetric studies (Dowsett *et al.*, 1989) have shown that a dose of 250 mg 2-weekly i.m. causes more variable suppression of oestradiol levels than 500 mg 2-weekly i.m. A recovery phenomenon was observed with oestradiol levels being significantly higher at 14 days than at 7 days. The recovery phenomenon was particularly seen in those with high (> 35 pmol $l^{-1}$) pretreatment levels.

In our study, apart from at week 1, hormone levels were only assessed after 14 days, just prior to the next injection. In view of the clinical response rate observed, a possible explanation is that oestrogen suppression was maximal between injections, recovery occurring before the next injection date. Levels at week 1 were not, however, lower than those at week 2, which does not support this hypothesis. Those with initially high oestradiol levels (>35 pmol l$^{-1}$, $n = 4$) appeared to have more suppression than those with lower initial levels, but this was not observed in those with high oestrone levels (>260 pmol l$^{-1}$, $n = 3$). The plasma concentration of 4-OHA following a single i.m. injection has previously been shown to fall gradually over a period of 28 days (Coombes et al., 1987), with a half-life of 8 days (Dowsett et al., 1984) suggesting that once fortnightly injections are sufficient. The long half-life is ascribed to the local formation of a drug depot following deep i.m. injection (Dowsett et al., 1989; data on file, Ciba Geigy). A dose of 250 mg i.m. fortnightly has previously been considered adequate in suppressing oestradiol levels (Coombes et al., 1987), It is not known to what level the (already very low) postmenopausal levels of oestrogens need to be further suppressed for clinical benefit. It is notable that despite a less than dramatic percentage fall in $E_1$ and $E_2$ levels, 7/18 (39%) of assessable patients achieved a partial response sustained for 8 months. It may be that tumour aromatase inhibition is more relevant than plasma oestrogen suppression (Kouyoumdjian et al., 1989; Hallam et al., 1989) and this aspect is currently being investigated. We feel, however, that further dosimetric studies should be carried out, as our data suggest that 4-OHA should be administered more frequently than 2-weekly in view of the suboptimal oestradiol and oestrone suppression.

SHBG reflects the oestrogen/androgen balance because the synthesis of SHBG is stimulated by oestrogen and inhibited by androgens. In postmenopausal women with very low oestrogen levels it is mainly a marker of androgenic activity. One patient developed increased facial hair, which might have been an androgenic side effect. However her SHBG level, which was high initially, rose further (105 to 120 nmol l$^{-1}$).

We have found a significant late fall of SHBG, occurring after 4 months on the drug from a mean of 87 to 45 nmol l$^{-1}$ (Figure 4). A fall in SHBG has also been shown following high dose oral treatment with 4-OHA (Dowsett et al., 1989), and has been explained by the rapid and high levels of drug presented to the liver – the site of SHBG synthesis, following oral absorption. This has not been previously noted following parenteral administration, even at higher dose, but the data previously available refers only to 15 patients followed for more than 1 month (Goss et al., 1986). Receptor studies in animals have shown 4-OHA to have approximately 1% of the androgenic activity of testosterone and androgenic activity has been confirmed in rats (Brodie et al., 1977). We have demonstrated that 4-OHA binds to SHBG with an association constant (ka) of $2 \times 10^8$ M$^{-1}$ (unpublished data at present). This implies that the drug does have weak androgenic activity (cf. testosterone ka = $8 \times 10^8$ M$^{-1}$; DHT ka = $10 \times 10^8$ M$^{-1}$).

The nature of our SHBG assay, based on the competitive binding of $^3$H-DHT to the binding protein is prone to potential interference. This potential interference can be due to the drug or its metabolites, either of which could support the possibility of the SHBG results being aberrantly low.

Three approaches can be taken to assess potential interference: (a) serum from 4-OHA treated patients can be treated with charcoal to remvoe potential interferants, but it is a problematic procedure, (b) measure SHBG levels before and after spiking with 4-OHA, however, this will not take account of any interfering metabolites or (c) the use of an immunoassay method for measurement of the SHBG protein directly, which will be independent of steroid interference. We took the last approach. SHBG levels were measured using the Farmos SHBG-IRMA kit (Pharmacia cat. no. 271001).

The Farmos SHBG-IRMA results were concordant with our saturation SHBG assay ($r = 0.936$; IRMA SHBG = 2.61 + 0.980 in house SHBG) confirming that our SHBG results were unaffected by competition of endogenous steroids in the patient samples.

DHAS, which acts as a marker of adrenal activity remained unchanged during the study. Cortisol levels did show an apparent fall between weeks 0 and 2, with subsequent improvement, but remained within the normal daytime range. The fall in cortisol may have been due to initially high stressed levels consequent upon the stress of the first outpatient visit, a stress that reduced as familiarity increased. All blood samples were taken between 10:00 and 12:00 – the temporal changes in serum cortisol levels are not significantly different during this time interval. There were no symptoms of adrenal insufficiency.

A possible alternative explanation is that 4-OHA has a direct effect upon adrenal steroidogenesis. This has not been previously noted in either animal or human studies (Goss et al., 1986; Coombes et al., 1984). However, although DHAS has been reportedly unchanged in 11 patients (Goss et al., 1986), cortisol levels were not reported in this trial. In one trial of only 11 patients (Coombes et al., 1984), cortisol levels were reportedly unchanged during the trial period. However, three of these patients died within one month of treatment. The effect of 4-OHA on cortisol levels is largely unknown and further investigations are necessary.

Aminoglutethimide (AG) is the most widely used aromatase inhibitor, but its main disadvantage is its toxicity. In our experience AG is frequently (44%) associated with systemic side effects such as rash (20%), lethargy and dizziness (9%). These effects are more common in the elderly and therapy has to be discontinued in 33% of those aged over 65 years (Rowell et al., 1987). More serious toxicity such as fatal agranulocytosis has occasionally been reported (Young et al., 1984; Vincent et al., 1985). In addition, AG has effects upon adrenal steroidogenesis, and should therefore be given with hydrocortisone supplements to prevent adrenal insufficiency (Murray & Pitt, 1985).

The side-effects that have been reported with 4-OHA include pain at the injection site, hot flushes, lethargy, rash, rarely anaphylaxis, transient leucopenia and facial swelling (Coombes et al., 1987). Our experience is similar, with one patient refusing further treatment, possibly as a result of developing a sterile abscess at the injection site, one possible allergic reaction, and a further five patients developing transient soreness at the injection sites. The incidence of symptomatic sterile abscesses appears dose related, varying from 6.4% (this study) with 250 mg to 13% with 50 mg (Coombes et al., 1987). Most patients develop an asymptomatic painless subcutaneous lump which resolves over a few weeks.

Although oral 4-OHA has been used, with encouraging clinical results, the parenteral route is currently favoured as the oral preparations of the drug are still in the early stages of development and evaluation. There appears to be marked variation in enteral absorption and hepatic first-pass, leading to a ten-fold difference in serum 4-OHA levels (Dowsett et al., 1989).

### *Conclusion*

Our results confirm the clinical effectiveness of 4-OHA. We report a RR of 39% with a mean duration of response of 8 months. An encouraging finding, also noted previously is that patients may respond to 4-OHA who have failed other hormonal manoeuvres.

The drug is better tolerated than AG, the only established aromatase inhibitor in clinical use. Its effect appears to be mediated by oestradiol and oestrone suppression. Our findings of a fall in SHBG level need to be further investigated, as this may indicate androgenic activity not previously reported with the drug.

Acknowledgments are due to Ciba-Geigy Pharmaceuticals for supplying 4-hydroxyandrostenedione (CGP 32349). Mr S. Hughes of Ciba Geigy Pharmaceuticals. Mrs P. Patel, Medical Statistician, Dr L. Dehenin (Oestrone Assay) and Miss T. Cocks for typing the manuscript.

**References**

BRODIE, A.M.H., COOMBES, R.C. & DOWSETT, M. (1987). Aromatase inhibitors: basic and clinical studies. *J. Steroid. Biochem.*, **27**, 899.

BRODIE, A.M., SCHWARZEL, W.C., SHAIKH, A.A. & BRODIE, H.J. (1977). The effect of an aromatase inhibitor – 4 Hydroxyandrostenedione on oestrogen dependent procedures in reproduction and breast cancer. *Endocrinology*, **100**, 1684.

COOMBES, R.C., GOSS, P.E., DOWSETT, M. & 4 others (1987). 4 Hydroxyandrostenedione treatment for postmenopausal patients with advanced breast cancer. *Tumor Diag. Ther.*, **8**, 271.

COOMBES, R.C., GOSS, P.E., DOWSETT, M., GAZET, J.-C. & BRODIE, A. (1984). 4-Hydroxyandrostenedione in treatment of postmenopausal patients with advanced breast cancer. *Lancet*, **ii**: 1237.

CUNNAH, D., JESSOP, D.S., BESSER, G.M. & REES, L.H. (1987). Measurement of circulating corticotrophin-releasing factor in man. *J. Endocrinol.*, **113**, 123.

CUNNINGHAM, D., POWLES, T.J., DOWSETT, M. & 5 others (1987). Oral 4-Hydroxyandrostenedione, a new endocrine treatment for disseminated breast cancer. *Cancer Chemother. Pharmacol.*, **20**, 253.

DOWSETT, M., GOSS, P.E., POWLES, T.J. & 4 others (1987). Use of the aromatase inhibitor 4-Hydroxyandrostenedione in postmenopausal breast cancer: optimisation of therapeutic dose and route. *Cancer Res.*, **47**, 1957.

DOWSETT, M., CUNNINGHAM, D.C., STEIN, R.C. & 4 others (1989). Dose-related endocrine effects and pharmacokinetics of oral and intramuscular 4 Hydroxyandrostenedione in post menopausal breast cancer patients. *Cancer Res.*, **49**, 1306.

FATTAH, D. & CHARD, T. (1981). Simplified method for measuring sex hormone binding globulin. *Clin. Chem.*, **27**, 1277.

GOSS, P.E., POWLES, T.J., DOWSETT, M. & 4 others (1986). Treatment of advanced postmenopausal breast cancer with an aromatase inhibitor, 4-Hydroxyandrostenedione: phase II report. *Cancer Res.*, **46**, 4823.

HALLAM, A., MANSEL, R.E., MILLER, W.R., NICHOLLS, P.J. & SMITH, H.J. (1989). Comparison of aminoglutethimide and pyridoglutethimide as aromatase inhibitors in breast tumour and adipose tissue *in vitro*. *Proc. Int. Soc. Breast Cancer Research*, Tel Aviv, (Abstr. B-32).

HOLLY, J.M.P., SMITH, C.P., DUNGER, D.B. & 7 others (1989). Relationship between the fall in sex-hormone binding globulin and insulin-like growth factor protein. A synchronised approach to pubertal development. *Clin. Endocrinol.*, **31**, 277.

KOUYOUMDJIAN, J.C., BEAUNE, J., RYMER, J.C. & FEUILHADE, F. (1989). Aromatase activity and hormono-sensitivity to aminoglutethimide in human breast carcinomas. *Proc. Int. Soc. Breast Cancer Research*, Tel Aviv, (Abstr. B-09).

MURRAY, P.A., PERRY, L., GILMORE, J. & PLOWMAN, P.N. (1988). 4 Hydroxyandrostenedione in advanced breast cancer: a phase 2 chemical endocrine study. *Proc. Am. Soc. Clin. Oncol.*, **7**, 17.

MURRAY, R. & PITT, P. (1985). Low dose aminoglutethimide without steroid replacement in the treatment of postmenopausal women with advanced breast cancer. *Eur. J. Cancer Clin. Oncol.*, **21**, 19.

PERRY, L.A., WATHEN, N. & CHARD, T. (1987). Saliva levels of oestradiol and progesterone in relation to non-protein-bound concentrations in blood during late pregnancy. *Hormone Metabol. Res.*, **19**, 444.

PERRY, L.A., AL-DUJAILI, E.A.S. & EDWARDS, C.R.W. (1982). A direct radioimmunoassay for 11-deoxycortisol. *Steroids*, **39**, 115.

REIFFSTECK, A., DEHENNIN, L. & SCHOLLER, R. (1982). Oestrogens in seminal plasma: identification and quantitative estimation by gas chromatography-mass spectrometry associated with stable isotope-dilution. *J. Steroid Biochem.*, **17**, 567.

ROWELL, N.P., GILMORE, O.J.A. & PLOWMAN, P.N. (1987). Aminoglutethimide as second line hormonal therapy in advanced breast cancer: response and toxicity. *Human Toxicol.*, **6**, 227.

SMITH, I.E., HARRIS, A.L., MORGAN, M., GAZET, J.-C. & MACKINNA, J.A. (1982). Tamoxifen vs Aminoglutethimide vs combined cancer. *Cancer Res.*, **42**, (suppl.), 3430.

STOLL, B.A. (1981). Breast cancer: rationale for endocrine therapy. In *Hormone Management of Endocrine-related Cancer*, Stoll, B.A. (ed.) p. 77. Lloyd-Luke: London.

VINCENT, M.D., CLINK, H.M., COOMBES, R.C. & 3 others (1985). Aminoglutethimide (with hydrocortisone) induced agranulocytosis in primary breast cancer. *Br. Med. J.*, **291**, 105.

WATHEN, N., PERRY, L.A., RUBENSTEIN, E. & CHARD, T. (1987). A relationship between sex hormone binding globulin and dihydroepiandrosterone sulphate in normally menstruating females. *Gynaecol. Endocrinol.*, **1**, 47.

WATHEN, N., PERRY, L., LILFORD, R.J. & CHARD. T. (1984). Interpretation of single progesterone measurement in diagnosis of anovulation and defective luteal phase: observations on analysis of the normal range. *Br. Med. J.*, **288**, 7.

YOUNG, J.A., NEWCOMBER, L.N. & KELLER, A.M. (1984). Aminoglutethimide-induced bone marrow injury. *Cancer*, **54**, 1731.